# Current Evidence on Traditional Chinese Exercise for Cancers: A Systematic Review of Randomized Controlled Trials

**DOI:** 10.3390/ijerph17145011

**Published:** 2020-07-12

**Authors:** Yang Song, Dong Sun, Bíró István, Anand Thirupathi, Minjun Liang, Ee-Chon Teo, Yaodong Gu

**Affiliations:** 1 Faculty of Sports Science, Ningbo University, Ningbo 315211, China; nbusongyang@hotmail.com (Y.S.); nbsundong@gmail.com (D.S.); ananthzeal@gmail.com (A.T.); liangminjun@nbu.edu.cn (M.L.); MECTEO@ntu.edu.sg (E.-C.T.); 2 Faculty of Engineering, University of Szeged, 6724 Szeged, Hungary; biro-i@mk.u-szeged.hu; 3 School of Chemical and Biomedical Engineering, Nanyang Technological University, Singapore 637459, Singapore

**Keywords:** cancer, traditional Chinese exercise, randomized controlled trial

## Abstract

Traditional Chinese exercise (TCE) has gradually become one of the widespread complementary therapies for treatment and recovery of cancers. However, evidence based on the systematic evaluation of its efficacy is lacking, and there appears to be no conclusion regarding the setting of TCE interventions. The purpose of this systematic review is to summarize the current randomized controlled trials (RCTs) that outline the effects of TCE on cancer patients. Relevant studies were searched by GOOGLE SCHOLAR, SCIENCEDIRECT, and WEB OF SCIENCE using “traditional Chinese exercise” and “cancer.” Only RCTs published in peer-reviewed English journals were included. A total of 27 studies covering 1616 cancer patients satisfied the eligibility criteria for this review. Despite the methodological limitation and relatively high risk of bias possessed by some included studies, positive evidence was still detected on the effects of TCE on these cancer-related health outcomes in physical, psychological, and physiological parameters. The 60-min or 90-min course of TCE intervention for two to three times per week for 10 to 12 weeks was found to be the most common setting in these studies and has effectively benefited cancer patients. These findings add scientific support to encourage cancer patients to practice TCE during or after conventional medical treatment. Nevertheless, future well-designed RCTs with improved methodology and larger sample size on this field are much warranted for further verification.

## 1. Introduction

Corona virus disease 2019 (COVID-19) has recently swept the globe, causing escalating health costs and economic loss to all countries in the world [1]. According to the latest report released by World Health Organization (WHO), coronavirus pneumonia has infected 3,917,366 people and caused 274,361 deaths worldwide by the time of 10:00 CEST, 10 May 2020 [2]. The situation now is the most severe in the United States, where the number of infection and death caused by COVID-19 is still rising rapidly [2]. Up to now, 1,300,696 cases of COVID-19 infection and 78,771 deaths have been confirmed in the United States based on the latest update from Centers for Disease Control and Prevention (CDC), accounting for almost a third of number worldwide [3].

While the COVID-19 has caused tremendous damage to human beings, cancer, as the second leading cause of death worldwide, has become one of the biggest threats to increase human life expectancy in the 21st century [4,5]. It is reported that cancer incidence and mortality have grown rapidly, with the number of cases increasing by 33% from 2005 to 2015 [6]. Besides, as a result of population aging and growth, the trend of diversification, complexity, and rejuvenation in cancer is also becoming more and more serious [4,7]. Based on the latest evaluate standard for estimating cancer incidence and mortality produced by the International Agency for Research on Cancer—GLOBOCAN 2018, Bray et al. [4] estimated that there were 18.1 million new confirmed cancer cases and 9.6 million deaths due to cancer in 2018 alone, which outnumber the figures reported in the COVID-19.

Despite the continuous development of cancer treatment over the past few decades, there are still considerable adverse effects in the standard therapies [8,9]. To be more specific, studies have found that the physical and psychological functions of cancer patients would all be deleteriously disturbed after several courses of chemotherapy [10,11]. Therefore, cancer patients are increasingly seeking for alternative therapies. In recent years, traditional Chinese exercise (TCE), such as Tai Chi Chuan and Qigong, has gained popularity all over the world. TCE generally refers to the mind-body exercise that emphasizes the inter-coordination of posture, breathing patterns, and meditation [12]. A growing number of studies have turned to examine the effects of TCE on cancer patients and most demonstrated that TCE enhances the efficacy of treatment and recovery of cancer [13,14]. However, there is still little evidence based on the systematic evaluation of their efficacy up to now, let alone conclusions regarding the setting of TCE interventions (i.e., mode, frequency, and duration) that may prove beneficial. Besides, some controversial results also exist, which made the efficacy of TCE a bit equivocal [15]. Thus, based on these motivations and the emerging benefits of TCE on cancers, it is of great significance to further address the above questions for cancer patients’ health promotion.

The purpose of this article is to review and summarize the current randomized controlled trials (RCTs) regarding the effects of TCE on cancer patients, find out the appropriate TCE intervention that can contribute to more beneficial outcomes for the treatment, and recovery of cancers, and give implications for future research.

## 2. Methods

### 2.1. Search Strategy and Data Sources

**Figure 1:**
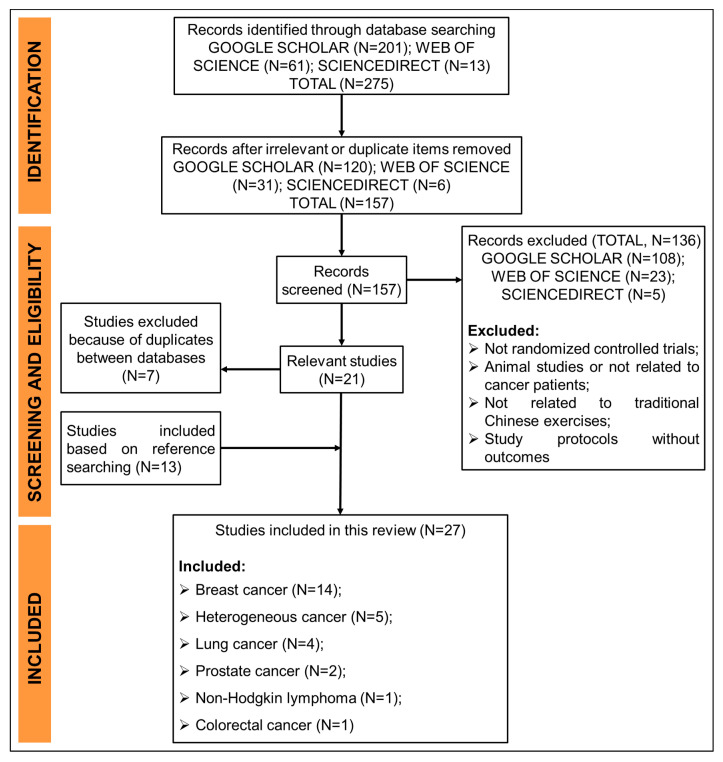
The search flowchart.

A thorough computer-aided literature search of GOOGLE SCHOLAR (all years), SCIENCEDIRECT (all years), and WEB OF SCIENCE (1960-present) was performed until 15 April 2020, to identify all relevant studies. The following search terms were used for this review “traditional Chinese exercise” and “cancer.” The search procedure is outlined in Figure 1, while the search strategy varies slightly with the above databases. In GOOGLE SCHOLAR database, the “advanced search” was applied, “traditional Chinese exercise” and “cancer” were put into “with all of the words” option and “anywhere in the article” was chosen. In SCIENCEDIRECT and WEB OF SCIENCE databases, the “keyword search” was chosen, and key words were entered in order: “traditional Chinese exercise” AND “cancer.”

To ensure the study searching process is thorough and rigorous, two authors independently checked and assessed all the retrieved records, and disagreements regarding inclusion (if happened) were resolved by discussing or consulting a third author. Furthermore, the citation snowballing method was used to manually locate all the potential relative papers in the bibliographies of the eligible articles or retrieved reviews [16,17].

### 2.2. Eligibility Criteria

The studies were eligible for inclusion if they met the following eligibility criteria: (1) Types of studies: only randomized controlled trials (RCTs) published in peer-reviewed English journals were covered. Observational studies, cross-sectional studies, reviews, case reports, conference papers were considered for exclusion. (2) Types of participants: patients who have been diagnosed with cancers were considered, but there is no restriction on age, gender, cancer type, tumor grade, and treatment state (pre-/mid-/post-treatment). (3) Types of interventions: Studies where TCE (e.g., Qigong, Tai Chi Chuan, and Baduanjin) was applied in the intervention group were included, but there is no restriction on the control group where usual care, low-intensity exercises, health education, or psychological therapy can be used. (4) Types of outcomes: Studies used physical, and/or psychological, and/or biochemical parameters to assess the effects of TCE on cancer patients were included.

### 2.3. Data Extraction and Management

For each study, the following data were extracted and summarized independently by an author and verified by another author, study characteristics (e.g., authors, nationality of the first author, and published year), participant characteristics (e.g., ethnicity, number of participants, age, gender, cancer types, and treatment state), description of interventions (e.g., exercise types, frequency, and duration), outcome parameters, and primary results. Disagreements (if any) were resolved by discussing or consulting a third author. Mendeley Reference Management Software (Elsevier Ltd., Amsterdam, The Netherlands) was applied for organizing papers and generating citations.

### 2.4. Quality Assessment

The quality of each included study was assessed independently by two authors based on the Cochrane Risk of Bias Assessment Tool [18]. A third author was consulted if any disagreements happened. The following seven domains were evaluated: random sequence generation, allocation concealment, blinding of participants and personnel, blinding of outcome assessment, incomplete outcome data, selective reporting, and other biases. Each domain has three grades: low risk of bias, unclear risk of bias, and high risk of bias.

## 3. Results

### 3.1. Search Results

As shown in Figure 1, the literature search yielded 275 records from the above three databases, and they were reduced to 157 after excluding all the irrelevant or duplicate studies. Then, according to the eligibility criteria, 108 papers from GOOGLE SCHOLAR, 23 papers from WEB OF SCIENCE, and 5 papers from SCIENCEDIRECT were further excluded for several reasons (e.g., studies were not randomized; animal studies or studies were not related to cancer patients; studies were not related to TCE; study protocols without any outcomes). Thirteen additional articles were identified after manually checking the reference lists of the eligible articles or retrieved reviews, while 7 studies were further excluded because of duplicates between databases. A total of 27 trials satisfied the eligibility criteria were finally included in this review.

### 3.2. Study Quality

**Figure 2:**
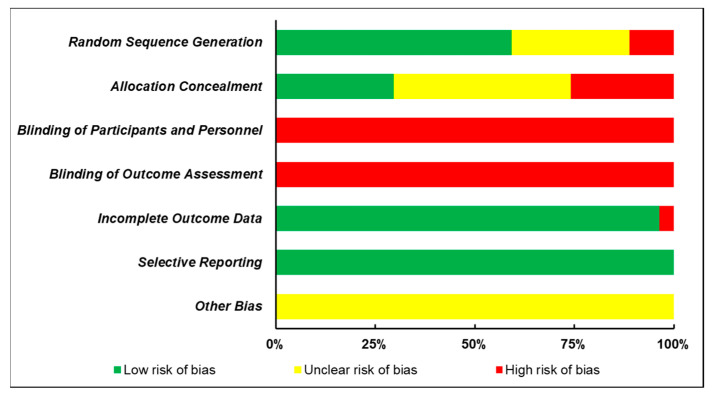
Risk of bias evaluation of included studies.

All included studies were assessed in terms of the risk of bias (Figure 2 and Table 1). Because of the nature of intervention, it may be not possible to blind the participants and/or personnel to group allocation and outcome. Therefore, the corresponding two domains, blinding of participants and personnel and blinding of outcome assessment, were the major sources of risk of bias from these studies (*n* = 27, 100.00%). In addition, of the 27 studies, only 16 trials (59.26%) described the method of group randomization in detail and 8 trials (29.63%) used the allocation concealment, which also increases the risk of bias. However, a low risk of incomplete outcome data bias (*n* = 26, 96.30%) and selective reporting bias (*n* = 27, 100.00%) was reported in most studies.

### 3.3. Basic Characteristics of Included Studies

The basic characteristics of all included studies are summarized in Table 2. The countries or regions of these publications are mainly the United States (*n* = 15, 55.56%), the People’s Republic of China (*n* = 8, 29.63%), Australia (*n* = 3, 11.11%), and Malaysia (*n* = 1, 3.70%). Twenty-seven studies covered 1616 cancer patients (14 studies covered patients with breast cancer; 5 studies covered patients with heterogeneous cancer; 4 studies covered patients with lung cancer; 2 studies covered patients with prostate cancer; 1 study covered patients with non-Hodgkin’s lymphoma; 1 study covered patients with colorectal cancer), and most of patients are Caucasian, Non-Latino, or White race. The modes of TCE intervention used in these studies were mostly Tai Chi and Qigong, and there are also two studies that combined the above two TCE. Of all the Tai Chi Chuan interventions applied in these studies, Yang-style Tai Chi Chuan was the most common one. On the other side, various types of Qigong were used, such as Guolin New Qigong, Chan-Chuang qigong, Zhi Neng qigong, and Baduanjin qigong. The total duration of TCE interventions varied from 3 to 24 weeks, with 10 to 12 weeks being the most common one. In addition, the intervention frequency ranged from 1 to 7 times per week, in which each time persisted for 20 to 120 min.

The primary findings that reported in these included studies are showed in Table 3. Summary of these results are presented in the following three parts: (1) Effects of TCE on physical outcomes; (2) effects of TCE on physiological outcomes; (3) effects of TCE on psychological outcomes.

#### 3.3.1. Effects of TCE on Physical Outcomes

Five studies that evaluated the effects of TCE on physical outcomes were included in this review. Galantino et al. [19] first started RCT on breast cancer patients. Subjects were required to take part in a 60-min course of Yang-style Tai Chi Chuan or walking interventions three times per week for 6 weeks in total. They found that both interventions have no appreciable effects on patients’ body mass index, and they indicated that it is due to the small sample size. Two subsequent articles also based on breast cancer patients reported some positive results [20,21]. Both trials employed a 12-week Yang-style Tai Chi Chuan or psychosocial interventions (60 min × 3 times/week) on subjects, and the results demonstrated that Tai Chi Chuan can improve the overall function capacity, including aerobic capacity, muscular strength, and flexibility, when compared to psychosocial therapy or pre-intervention.

Finally, two recent studies have investigated the effects of Qigong on cancer patients. Ying et al. [22] compared the effects of a continuous Baduanjin qigong intervention (60 min per day for 6 months) with original physical activity on breast cancer patients and found that the former have a significantly better effect on physical rehabilitation, such as body mass index and shoulder range of motion, which put a conflicting result with Galantino et al. study [19]. Lu et al. [23] conducted a relatively similar study on colorectal cancer patients. Subjects were asked to perform Baduanjin qigong exercise (20–40 min) five times per week for 24 weeks. They reported that this type of TCE can help improve physical activity level.

#### 3.3.2. Effects of TCE on Physiological Outcomes

Twelve studies were included in this category. Oh et al. conducted three trials in 2008, 2010, and 2012, respectively [24,25,26], with the aim to investigate the effects of Qigong on inflammatory markers in cancer patients (mainly breast cancer patients). A 90-min course of modified Qigong program was applied in their studies two times per week for 8 to 10 weeks, and all three studies demonstrated that Qigong exercise can significantly decrease the level of inflammatory markers compared to control group where subjects only received standard medical care. It is interesting to note that, Peppone et al. [14] in 2010 compared the influences of TCE with standard support therapy on bone loss biomarkers in breast cancer patients. They reported that a 12-week Yang-style Tai Chi Chuan intervention for three times per week with 60 min per time can exert more positive effects on bone health of breast cancer patients. In 2011 and 2014, similar to what Oh et al. found, two studies examined the influences of Tai Chi Chuan exercise on inflammatory markers in breast cancer patients and also reported positive effects [27,28]. Twelve-week Yang-style Tai Chi Chuan intervention (60 min × 3 times/week) and 3-month Tai Chi Chuan intervention (120 min per week) were applied in the two studies, respectively. Besides the effects of TCE on inflammatory markers, the expression of genes encoding pro-inflammatory mediators was found lower after TCE by Irwin et al. [27], while Janelsins et al. [28] also reported that the level of insulin remained stable after TCE but increased after control intervention when compared to pre-intervention. However, conflicting results also emerged in recent years. Although there are some differences in the TCE intervention settings (three times per week at 60 min per time for 12 weeks by Campo et al. [29], while 90 min per week for 10 weeks by Sprod et al. [30]), these two studies reported that Tai Chi Chuan intervention exhibited no superior effects on inflammatory markers than control group. Nevertheless, Campo et al. [29] found some positive effects on blood pressure and salivary cortisol after TCE intervention. Also, in terms of blood parameters, in a larger RCT in 2017, Chuang et al. [13] investigated the effects of 21-day Chan-Chuang Qigong intervention (25 min per time for 2 to 3 times per day) on 100 non-Hodgkin lymphoma patients, and they reported that the white blood cell counts and hemoglobin levels were significantly improved after TCE when compared to control intervention.

There are three more studies included in this category, which evaluated the effects of 16-week Tai Chi Chuan intervention (60 min per time for three times per week), respectively on the proliferation and cytolytic/tumoricidal activities of peripheral blood mononuclear cell [31], the balance between cellular and humoral immunity [32], and the cellular immune responses [33] in non-small cell lung cancer patients. All of them found positive, statistically significant effects after TCE intervention.

#### 3.3.3. Effects of TCE on Psychological Outcomes

The majority of RCTs included in this review (19 out of 27 trials) investigated the effects of TCE on psychological outcomes in cancer patients. To be more specific, quality of life was assessed in 11 studies, fatigue in 10 studies, mood status (e.g., distress, anxiety, and depression) in 8 studies, sleep quality in 6 studies, and cognitive function in 1 study.

The Functional Assessment of Cancer Therapy-General (FACT-G) was generally used to measure the score of quality of life. Of all these 11 studies, most (*n* = 9) reported significantly positive effects after TCE interventions on quality of life, and the 60 or 90 min per time at 2–3 times per week for 10–12 weeks was the commonly used setting of TCE intervention [13,21,24,25,26,30,34,35,36]. Nevertheless, the remaining two studies conducted in 2013 proposed some different results. Campo et al. [37] employed a 12-week Tai Chi Chuan intervention (60 min per time for three times per week) on solid tumor cancer patients (83% breast cancer patients), and they reported that there is no difference on quality of life between TCE and control intervention. Robins et al. [38] also found no difference between groups and even compared to pre-intervention after 10-week Tai Chi Chuan intervention (90 min per week) on breast cancer patients. Assessed using Brief Fatigue Inventory (BFI) or Functional Assessment of Chronic Illness Therapy (FACIT), more than half studies (seven of ten trials) found that TCE can significantly reduce the cancer-related fatigue [13,23,25,34,39,40,41]. However, the setting of TCE intervention varied greatly between studies, which make it inconclusive. Besides, contrary findings also existed. Two studies based on breast cancer patients and one study on prostate cancer patients reported no positive effects after 6-week Yang-style Tai Chi Chuan intervention (60 min per time for three times per week) [19], 8-week Zhi Neng Qigong intervention (30 min per time for three times per week) [35], and 6- to 8-week Yang-style Tai Chi Chuan and Qigong intervention (40 min per time for three times per week) [42], respectively. In terms of mood status and sleep quality, conflicting and equivocal results continued to emerge in recent years. Five of eight studies that examined the effects of TCE on mood status using corresponding self-administered questionnaire (e.g., Profile of Mood State (PMS)) reported significantly positive effects compared to control intervention [22,25,34,36,39], while the remaining three studies found no difference [35,38,40]. Similarly, only two of six studies that investigated the effects of TCE on sleep quality using corresponding self-rated questionnaire (e.g., Pittsburgh Sleep Quality Index (PSQI)) reported significantly positive effects compared to control intervention [13,23], while the remaining four studies found no difference [34,40,42,43]. The diversified settings of TCE intervention may be the potential reason for these contraries. Finally, a study by Oh et al. [26] explored the effects of a 90-min modified Qigong program (two times per week for 10 weeks) on cognitive function in patients with heterogeneous cancers, and the results showed that TCE was more effective in improving cognitive function than the control intervention.

## 4. Discussion

This systematic review comprehensively summarized evidence from a large number of RCTs investigating the effects of TCE on health outcomes in cancer patients during or after the standard medical treatment, with the aim to determine the appropriate setting of TCE intervention that can contribute to beneficial outcomes for the treatment and recovery of cancer.

There is a great variability in outcome parameters reported in these included studies. To be more specific, physical, physiological, and psychological parameters are all examined in the 27 trials covered in this review. Regarding physical and physiological outcomes, most of the related studies reported significantly positive effects after TCE interventions. Nevertheless, one study investigated the effect of TCE on body mass index and two on inflammatory markers have not come out with better results when compared to pre-intervention or control group. Galantino et al. [19] demonstrated that the small sample size may be the main reason for this controversy. Findings became more conflicting and equivocal when it comes to psychological parameters, which may be due to the limited number of studies after they have been divided into several secondary outcomes, such as quality of life, fatigue, mood status, sleep quality, and cognitive function. In terms of the TCE intervention, Tai Chi and Qigong have been widely used in these studies, and a thorough examination of the included trials indicated that the 60 or 90 min per time at two to three times per week for 10 to 12 weeks is the most common and frequent used setting. Overall, the findings of this review added support to the previous studies suggested that cancer patients may benefit more from TCE both during and after the standard medical treatment. However, more RCTs investigating the effects of TCE intervention on the psychological outcomes in cancer patients are much warranted for further verification.

The potential mechanisms by which TCE can help in the treatment and recovery of cancer patients have been extensively speculated, while data compiled in this review showed only restricted evidence to support these. TCE is a complex mind-body exercise that is based on the essential theories of Chinese medicine [12]. Most forms focus on the inter-coordination of posture, breathing patterns, and meditation, by which the natural health recovery-mechanisms could be evoked, while the release level of endogenous neurohormones could be balanced [44], thus, the physical and emotional tension and immune function may be improved [25]. It is interesting to note that a study by Janelsins et al. [28] developed a model to compare the micro-molecular changes that occurred during TCE or non-physical activity interventions in breast cancer patients. They hypothesized that cytokines interleukin (IL-6) may be associated with the inflammation-medicated cellular proliferation and tumor growth, while with the processing of TCE intervention (e.g., Tai Chi Chuan), the derived IL-6 would contribute to the anti-inflammatory effects and the lipolysis then is induced, which ultimately help in maintaining weight and reducing the recurrence risk. Also, studies have demonstrated that overweight cancer patients (e.g., breast, prostrate, and colorectal cancer patients) may be associated with higher risk of cancer recurrence and even death [45,46,47]. Thus, the above internal mechanism could be plausible to some specific cancer patients. Nevertheless, the underlying mechanisms may vary with many aspects (e.g., population, the type, and stage of cancer) and remain to be further clarified.

Some notable flaws of study design that existed in these included studies need further addressing. For example, small sample size is problematic as it may increase the risk of type Π error for results [48]. Of the 27 included studies, more than one-third of the studies (*n* = 12) had a sample size of less than 50, with the smallest number being 11 participants. Thirteen studies were based on a sample size of more than 50 but less than 100. Only 2 studies covered a sample of more than 100. In addition, the relatively high risk of bias possessed by these included studies is another problem. As stated in the result part, blinding of participants and personnel and blinding of outcome assessment were the major sources of risk of bias from these studies. Besides, of the 27 included studies, only 16 studies described the method of group randomization in detail and 8 used the allocation concealment. Thus, the result reliability of these studies would be weakened, which may also put certain influence on the synthesis of these results.

This review also brings some implications for future studies on this field. Above all, future research needs to investigate the long-term impacts of TCE intervention in a large sample. The motivation and exercise history of the recruited patients should be assessed before group randomization in order to maximize their adherence to the TCE programs. In addition, most included studies explored the effect of Tai Chi or Qigong on cancer patients. However, other TCE modalities, such as Yijinjing and Liuzijue, are also worth studying.

## 5. Conclusions

In conclusion, this review further confirmed that TCE could be more beneficial than the standard medical care or even other exercises with the similar intensity for improving physical, physiological, and psychological outcomes in cancer patients. The 60-min or 90-min course of TCE intervention for two to three times per week for 10 to 12 weeks was found to be the most common setting in these studies that may effectively benefit cancer patients. However, limited number of RCTs in certain field (e.g., psychology), methodological flaws, and relatively high risk of bias in these included studies remain to be further addressed and clarified.

## Figures and Tables

**Table 1:** Risk of bias evaluation of included studies.

Trials	Random Sequence Generation	Allocation Concealment	Blinding of Participants and Personnel	Blinding of Outcome Assessment	Incomplete Outcome Data	Selective Reporting	Other Bias
Campo et al. (2013) [37]	Unclear	Unclear	High	High	Low	Low	Unclear
Campo et al. (2014) [39]	Unclear	Unclear	High	High	Low	Low	Unclear
Campo et al. (2015) [29]	Low	Unclear	High	High	Low	Low	Unclear
Chen et al. (2013) [34]	Low	Unclear	High	High	Low	Low	Unclear
Chuang et al. (2017) [13]	Low	Low	High	High	Low	Low	Unclear
Galantino et al. (2003) [19]	Low	High	High	High	High	Low	Unclear
Irwin et al. (2014) [27]	Unclear	Unclear	High	High	Low	Low	Unclear
Irwin et al. (2017) [43]	Unclear	Low	High	High	Low	Low	Unclear
Janelsins et al. (2011) [28]	Low	Low	High	High	Low	Low	Unclear
Larkey et al. (2014) [40]	Low	Low	High	High	Low	Low	Unclear
Liu et al. (2015) [31]	Low	High	High	High	Low	Low	Unclear
Loh et al. (2014) [35]	Low	Low	High	High	Low	Low	Unclear
Lu et al. (2019) [23]	Unclear	Unclear	High	High	Low	Low	Unclear
McQuade et al. (2017) [42]	Low	Low	High	High	Low	Low	Unclear
Mustian et al. (2004) [36]	Unclear	High	High	High	Low	Low	Unclear
Mustian et al. (2006) [20]	Low	High	High	High	Low	Low	Unclear
Mustian et al. (2008) [21]	Unclear	Unclear	High	High	Low	Low	Unclear
Oh et al. (2008) [24]	High	High	High	High	Low	Low	Unclear
Oh et al. (2010) [25]	High	High	High	High	Low	Low	Unclear
Oh et al. (2012) [26]	High	Unclear	High	High	Low	Low	Unclear
Peppone et al. (2010) [14]	Low	High	High	High	Low	Low	Unclear
Robins et al. (2013) [38]	Low	Unclear	High	High	Low	Low	Unclear
Sprod et al. (2012) [30]	Low	Low	High	High	Low	Low	Unclear
Wang et al. (2013) [32]	Unclear	Unclear	High	High	Low	Low	Unclear
Ying et al. (2019) [22]	Low	Unclear	High	High	Low	Low	Unclear
Zhang et al. (2013) [33]	Low	Unclear	High	High	Low	Low	Unclear
Zhang et al. (2016) [41]	Low	Low	High	High	Low	Low	Unclear

**Table 2:** The study characteristics of included studies.

Trial	Country /Region	Cancer Type	Ethnicity	Treatment State	Sample Size (N)	Gender and Age (Year)	Exercise Intervention
Campo et al. (2013) [37]	USA	Solid tumor cancer (breast cancer 83%)	TCE Non-Latino 97%, Latino 3% CON Non-Latino 94%, Latino 4%	Treatment completed (≥3 months)	N = 63 TCE = 32 CON = 31	Gender TCE, F = 32 CON, F = 31 Age TCE, 66.54 (55–89) CON, 65.64 (57–84)	TCE Tai Chi Chih, 60-min × 3 times/week, 12 weeks CON Health education, 60-min × 3 times/week, 12 weeks
Campo et al. (2014) [39]	USA	Prostate cancer	TCE Non-Latino 100% CON Non-Latino 92%, Latino 8%	NA	N = 29 TCE = 16 CON = 13	Gender TCE, M = 16 CON, M = 13 Age TCE, 72 (58–90) CON, 73 (61–93)	TCE Qigong, 60-min × 2 times/week, 12 weeks CON Nonaerobic stretching exercise, 60-min × 2 times/week, 12 weeks
Campo et al. (2015) [29]	USA	Solid tumor cancer (breast cancer 80%)	TCE Non-Latino 93%, White race 97% CON Non-Latino 96%, White race 100%	Treatment completed (≥3 months)	N = 54 TCE = 29 CON = 25	Gender TCE, F = 29 CON, F = 25 Age TCE, 65.9 (55–82) CON, 66.7 (59–84)	TCE Tai Chi Chih, 60-min × 3 times/week, 12 weeks CON Health education, 60-min × 3 times/week, 12 weeks
Chen et al. (2013) [34]	China	Breast cancer	NA	Undergoing treatment	N = 96 TCE = 49 CON = 47	Gender TCE, F = 49 CON, F = 47 Age TCE, 45.3 ± 6.3; CON, 44.7 ± 9.7	TCE Guolin New Qigong, 40-min × 5 times/week, 5–6 weeks CON NA
Chuang et al. (2017) [13]	Taiwan, China	Non-Hodgkin lymphoma	NA	After the first course of treatment	N = 96 TCE = 48 CON = 48	Gender TCE, M = 26, F = 22 CON, M = 29, F = 19 Age TCE, 55.85 ± 16.78 CON, 64.54 ± 15.51	TCE Chan-Chuang qigong, 25-min × 2 to 3 times/day, 21 days CON NA
Galantino et al. (2003) [19]	USA	Breast cancer	NA	Treatment completed in the past year	N = 11 TCE = 6 CON = 5	Gender TCE, F = 6 CON, F = 5 Age 40–59	TCE Yang-style Tai Chi Chuan, 60-min × 3 times/week, 6 weeks CON Walking intervention, 60-min × 3 times/week, 6 weeks
Irwin et al. (2014) [27]	USA	Breast cancer	TCE White race 75.6% CON White race 95.6%	Treatment completed (≥6 months)	N = 90 TCE = 45 CON = 45	Gender TCE, F = 45 CON, F = 45 Age TCE, 59.6 ± 7.9 CON, 60.0 ± 9.3	TCE Tai Chi Chih, 120-min per week, 3 months CON Cognitive behavioral therapy for insomnia, 120-min per week, 3 months
Irwin et al. (2017) [43]	USA	Breast cancer	TCE White race 75.6% CON White race 95.6%	Treatment completed (≥6 months)	N = 90 TCE = 45 CON = 45	Gender TCE, F = 45 CON, F = 45 Age TCE, 59.6 ± 7.9 CON, 60.0 ± 9.3	TCE Tai Chi Chih, 120-min per week, 3 months CON Cognitive behavioral therapy for insomnia, 120-min per week, 3 months
Janelsins et al. (2011) [28]	USA	Breast cancer	TCE White race 100% CON White race 100%	Treatment completed (≥1 month and ≤30 months)	N = 19 TCE = 9 CON = 10	Gender TCE, F = 9 CON, F = 10 Age TCE, 54.33 ± 10.64 CON, 52.70 ± 6.67	TCE Yang-style Tai Chi Chuan, 60-min × 3 times/week, 12 weeks CON Psychosocial support therapy, 60-min × 3 times/week, 12 weeks
Larkey et al. (2014) [40]	USA	Breast cancer	TCE Latino 2.38%, Non-Latino 83.33% CON Latino 2.22%, Non-Latino 88.89%	Treatment completed (≥6 months and ≤5 years)	N = 87 TCE = 42 CON = 45	Gender TCE, F = 42 CON, F = 45 Age TCE, 57.7 ± 8.94 CON, 59.8 ± 8.93	TCE Qigong/Tai Chi Easy, 30-min × 5 times/week, 12 weeks CON Sham Qigong, 30-min × 5 times/week, 12 weeks
Liu et al. (2015) [31]	China	Non-small cell lung cancer	NA	Treatment completed (≥2 years)	N = 27 TCE = 14 CON = 13	Gender TCE, M = 8, F = 6 CON, M = 7, F = 6 Age TCE, 62.64 ± 8.35 CON, 60.46 ± 7.08	TCE Yang-style Tai Chi Chuan, 60-min × 3 times/week, 16 weeks CON Hospital care
Loh et al. (2014) [35]	Malaysia	Breast cancer	TCE Chinese 59.4%, Malay 31.3%, Indian 3.2% CON 1 Chinese 71.0%, Malay 19.4%, Indian 9.7% CON 2 Chinese 62.5%, Malay 25%, Indian 12.1%	Treatment completed (≥1 years)	N = 95 TCE = 32 CON1 = 31 CON2 = 32	Gender TCE, F = 32 CON1, F = 31 CON2 = 32 Age 18–65	TCE Zhi Neng Qigong, 30-min × 3 times/week, 8 weeks CON1 A group line-dancing program, 30-min × 3 times/week, 8 weeks CON2 Standard medical care
Lu et al. (2019) [23]	China	Colorectal cancer	NA	Undergoing treatment	N = 87 TCE = 43 CON = 44	Gender TCE, M = 26, F = 17 CON, M = 30, F = 14 Age TCE, 55.60 ± 11.23 CON, 54.63 ± 11.88	TCE Baduanjin, (20–40)-min × 5 times/week, 24 weeks CON Health education
McQuade et al. (2017) [42]	USA	Prostate cancer	TCE White 95.2%, Hispanic 4.8% CON 1 White 85.7%, Hispanic 9.5%, Asian 4.8% CON 2 White 95.8%, Black 4.2%	Undergoing treatment	N = 66 TCE = 21 CON1 = 21 CON2 = 24	Gender TCE, M = 21 CON1, M = 21 CON2, M = 24 Age TCE, 62.2 ± 7.4 CON1, 65.0 ± 5.9 CON2, 66.0 ± 8.4	TCE Yang-style Tai Chi and Qigong, 40-min × 3 times/week, 6–8 weeks CON1 Light resistance training and stretching exercise, 40-min × 3 times/week, 6–8 weeks CON2 NA
Mustian et al. (2004) [36]	USA	Breast cancer	Caucasian 90%	Treatment completed (≥1 week and ≤30 months)	N = 21 TCE = 11 CON = 10	Gender TCE, F = 11 CON, F = 10 Age 52 ± 9	TCE Yang-style Tai Chi Chuan, 60-min × 3 times/week, 12 weeks CON Psychosocial support, 60-min × 3 times/week, 12 weeks
Mustian et al. (2006) [20]	USA	Breast cancer	Caucasian 90%	Treatment completed (≥1 week and ≤30 months)	N = 21 TCE = 11 CON = 10	Gender TCE, F = 11 CON, F = 10 Age 52 ± 9	TCE Yang-style Tai Chi Chuan, 60-min × 3 times/week, 12 weeks CON Psychosocial therapy, 60-min × 3 times/week, 12 weeks
Mustian et al. (2008) [21]	USA	Breast cancer	Caucasian 90%	Treatment completed (≥1 week and ≤30 months)	N = 21 TCE = 11 CON = 10	Gender TCE, F = 11 CON, F = 10 Age 52 ± 9	TCE Yang-style Tai Chi Chuan, 60-min × 3 times/week, 12 weeks CON Psychosocial support, 60-min × 3 times/week, 12 weeks
Oh et al. (2008) [24]	Australia	Breast cancer (40%); Ovary cancer (20%) etc.	Caucasian 83%, Asian 10%, Indigenous Australian 7%	NA	N = 30 TCE = 15 CON = 15	Gender TCE, M = 3, F = 12 CON, M = 3, F = 12 Age 54 ± 9	TCE Modified Qigong program, 90-min × 1–2 times/week, 8 weeks CON Usual care
Oh et al. (2010) [25]	Australia	Breast cancer (34%), colorectal cancer (12%) etc.	TCE Caucasian 77.0%, Asian 13.5%, Indigenous Australian 1.4%, Other 8.1% CON Caucasian 64.5%, Asian 22.4%, Indigenous Australian 1.3%, Other 11.8%	NA	N = 162 TCE = 79 CON = 83	Gender TCE, M = 31, F = 48 CON, M = 38, F = 45 Age TCE, 60.1 ± 11.7 CON, 59.9 ± 11.3	TCE Modified Qigong program, 90-min × 2 times/week, 10 weeks CON Usual care
Oh et al. (2012) [26]	Australia	Breast cancer, colorectal cancer etc.	TCE Caucasian 82.4%, Asian 11.8% CON Caucasian 57.5%, Asian 22.5%	received or undergoing treatment	N = 76 TCE = 36 CON = 40	Gender TCE, M = 18, F = 18 CON, M = 20, F = 20 Age TCE, 64.6 ± 12.3 CON, 61.1 ± 11.0	TCE A modified Qigong program, 90-min × 2 times/week, 10 weeks CON Usual care
Peppone et al. (2010) [14]	USA	Breast cancer	TCE White race 100% CON White race 100%	Treatment completed (≥1 month and ≤30 months)	N = 16 TCE = 7 CON = 9	Gender TCE, F = 7 CON, F = 9 Age TCE, 53.8 (Average) CON, 52.6 (Average)	TCE Yang-style Tai Chi Chuan, 60-min × 3 times/week, 12 weeks CON Standard support therapy, 60-min × 3 times/week, 12 weeks
Robins et al. (2013) [38]	USA	Breast cancer	Caucasian 75%, African American 25%	Undergoing treatment	N = 109	Gender F = 109 Age 50 (Average, 27–75)	TCE Tai Chi, 90-min per week, 10 weeks CON1 Personal exploration and group sharing of spirituality, 90-min per week, 10 weeks CON2 Usual care
Sprod et al. (2012) [30]	USA	Breast cancer	TCE White race 100% CON White race 100%	Treatment completed (≥1 month and ≤30 months)	N = 19 TCE = 9 CON = 10	Gender TCE, F = 9 CON, F = 10 Age TCE, 54.33 ± 3.55 CON, 52.70 ± 2.11	TCE Yang-style Tai Chi Chuan, 60-min × 3 times/week, 12 weeks CON Psychosocial therapy, 60-min × 3 times/week, 12 weeks
Wang et al. (2013) [32]	China	Non-small lung cancer	NA	Treatment completed (≥2 years)	N = 27 TCE = 13 CON = 14	Gender TCE, M = 7, F = 6 CON, M = 8, F = 6 Age TCE, 63.1 ± 7.9 CON, 59.3 ± 7.7	TCE Tai Chi, 60-min × 3 times/week, 16 weeks CON NA
Ying et al. (2019) [22]	China	Breast cancer	NA	Treatment completed (≤2 years)	N = 86 TCE = 46 CON = 40	Gender TCE, F = 46 CON, F = 40 Age 54.09 ± 7.76	TCE Baduanjin exercise (Qigong), 60-min per day, 6 months CON Original physical activity, no less than 30-min per day, 6 months
Zhang et al. (2013) [33]	China	Non-small cell lung cancer	NA	Treatment completed (≥2 years)	N = 27 TCE = 14 CON = 13	Gender TCE, M = 8, F = 6 CON, M = 7, F = 6 Age TCE, 63.07 ± 7.89 CON, 59.27 ± 7.68	TCE Tai Chi Chuan, 60-min × 3 times/week, 16 weeks CON NA
Zhang et al. (2016) [41]	China	Lung cancer	NA	Receiving 2–4 treatment courses for a 21-day cycle	N = 91 TCE = 47 CON = 44	Gender TCE, M = 37, F = 10 CON, M = 31, F = 13 Age 62.8 (Average)	TCE Simplified Yang-style Tai Chi, 60-min × every other day, 12 weeks CON Low-impact exercise, 60-min × every other day, 12 weeks

Note: control intervention, CON; female, F; not available, NA; number, N; male, M; traditional Chinese exercise, TCE.

**Table 3:** The main results of included studies.

Trials	Outcome Parameters	Primary Results
Campo et al. (2013) [37]	Quality of life.	Although mental score after TCE is higher than pre-intervention, there is no difference in quality of life between the two groups.
Campo et al. (2014) [39]	Fatigue; Distress.	Less fatigue and distress after TCE compared with CON.
Campo et al. (2015) [29]	Blood pressure; Salivary cortisol; Inflammatory cytokines.	Lower systolic blood pressure and cortisol area-under-curve after TCE compared to CON; No difference between interventions on inflammatory cytokines.
Chen et al. (2013) [34]	Depressive symptoms; Fatigue; Sleep disturbances; Quality of life.	Less depressive symptoms after TCE compared to CON; Improvement in overall quality of life and less fatigue in subjects with elevated depressive symptoms during treatment after TCE compared to CON. No difference in sleep disturbances.
Chuang et al. (2017) [13]	Fatigue; Complete blood cells; Sleep quality; Quality of life.	Fatigue intensity and interference were reduced after TCE compared to CON; Improvement in white blood cell counts and hemoglobin levels after TCE compared to CON; Improvement in sleep quality and quality of life after TCE compared to CON.
Galantino et al. (2003) [19]	Fatigue; Body mass index.	All interventions have no appreciable effects on these parameters.
Irwin et al. (2014) [27]	Cellular inflammation; Transcriptome dynamics.	Lower cellular inflammatory responses and expression of genes encoding pro-inflammatory mediators after TCE compared to CON.
Irwin et al. (2017) [43]	Insomnia treatment response; Insomnia remission.	Improvement in insomnia after both groups, but no difference between the two groups.
Janelsins et al. (2011) [28]	Inflammatory cytokines; Insulin.	Improvement in the level of cytokine/myokine IL-6 after TCE compared to CON; The levels of insulin remained stable after TCE but increased after CON compared to pre-intervention.
Larkey et al. (2014) [40]	Fatigue; Depression; Sleep.	Less fatigue after TCE compared to CON but not for depression and sleep quality; Improvement in depression and sleep quality after both group compared to pre-intervention.
Liu et al. (2015) [31]	The proliferation and cytolytic/tumoricidal activities of peripheral blood mononuclear cell.	Improvement in peripheral blood mononuclear cell proliferation and cytolytic/tumoricidal activities of peripheral blood mononuclear cell against A549 cells after TCE compared to CON.
Loh et al. (2014) [35]	Quality of life; Distress; Fatigue.	Improvement in quality of life after TCE compared to the other two groups; No significant difference in distress and fatigue between groups.
Lu et al. (2019) [23]	Fatigue; Physical activity level; Sleep quality.	Lower proportion of patients with moderate-to-severe cancer-related fatigue after TCE compared to CON; Improvement in physical activity level and sleep quality after TCE compared to CON.
McQuade et al. (2017) [42]	Sleep disturbances; Fatigue.	Longer sleep duration at mid-intervention of TCE compared with the other two groups, but no difference after interventions; No difference in other parameters of sleep and fatigue.
Mustian et al. (2004) [36]	Quality of life; Self-esteem.	Improvement in quality of life and self-esteem after TCE compared to CON.
Mustian et al. (2006) [20]	Function capacity.	Improvement in functional capacity (aerobic capacity, muscle strength, and flexibility) after TCE compared to pre-intervention; Improvement only in flexibility after CON compared to pre-intervention.
Mustian et al. (2008) [21]	Functional capacity; Quality of life.	Improvement in functional capacity (aerobic capacity, muscular strength, and flexibility) and quality of life after TCE compared to pre-intervention; Improvement in flexibility while decline in aerobic capacity, muscular strength, and quality of life after CON compared with pre-intervention.
Oh et al. (2008) [24]	Quality of life; Symptoms and side effects of cancer; Inflammation biomarkers	Improvement in quality of life after TCE compared to pre-intervention; Inflammation biomarker, symptoms and side effects of cancer were reduced after TCE compared to CON but without significance.
Oh et al. (2010) [25]	Quality of life; Fatigue; Mood status; Inflammatory markers.	Improvement in overall quality of life, fatigue, mood status, and inflammatory marker serum C-reactive protein after TCE compared to CON.
Oh et al. (2012) [26]	Cognitive function; Quality of life; Inflammation biomarkers.	Improvement in cognitive function and quality of life after TCE compared to CON; Lower C-reactive protein levels after TCE compared to CON.
Peppone et al. (2010) [14]	Bone loss biomarkers.	Improvement in the levels of bone formation after TCE compared to CON; Lower levels of bone resorption after TCE compared to CON.
Robins et al. (2013) [38]	Psychosocial functioning; Quality of life; Biological markers.	All interventions have no appreciable effects on these parameters.
Sprod et al. (2012) [30]	Quality of life.	Improvement in overall quality of life after TCE, while improved in part of quality of life after CON compared to pre-intervention.
Wang et al. (2013) [32]	The balance between cellular and humoral immunity.	The balance between cellular and humoral immunity remained stable after TCE compared to CON.
Ying et al. (2019) [22]	Physical parameters (e.g., body mass index, shoulder range of motion etc.); Psychological parameters (e.g., anxiety, depression etc.).	Improvement in both physical and psychological parameters after TCE compared to CON.
Zhang et al. (2013) [33]	Cellular immune responses.	Lower increment in complement regulatory proteins status CD55 expression after TCE compared to CON.
Zhang et al. (2016) [41]	Fatigue.	Less fatigue after TCE compared to CON.

Note: control intervention, CON; traditional Chinese exercise, TCE.
